# Complete Genome Sequence of *Mycobacterium* sp. MS1601, a Bacterium Performing Selective Oxidation of Polyols

**DOI:** 10.1128/genomeA.00156-17

**Published:** 2017-04-13

**Authors:** Mahmoud Sayed, Waiel F. Sayed, Rajni Hatti-Kaul, Sang-Hyun Pyo

**Affiliations:** a Division of Biotechnology, Department of Chemistry, Center for Chemistry and Chemical Engineering, Lund University, Lund, Sweden; b Department of Botany, Faculty of Science, South Valley University, Qena, Egypt

## Abstract

*Corynebacterium* sp. (ATCC 21245) is reclassified here as *Mycobacterium* sp. MS1601 based on 16S rRNA gene and complete-genome sequence analysis. It is able to oxidize branched polyols to corresponding hydroxycarboxylic acids. The total size of the genome sequence was 6,829,132 bp, including one circular chromosome of 6,407,860 bp.

## GENOME ANNOUNCEMENT

*Mycobacterium* spp., most frequently isolated from soil, are aerobic, Gram-positive, acid-fast bacteria that are slightly curved or have a straight rod shape (1, 2). In this study, *Corynebacterium* sp. (ATCC 21245) is reclassified as *Mycobacterium* sp. MS1601 based on 16S rRNA gene and complete-genome sequence analysis. *Corynebacterium* sp. (ATCC 21245) has been deposited and classified as a nonpathogenic strain by ATCC.

Recently, we reported that *Corynebacterium* sp. (ATCC 21245) carries out selective oxidation of branched polyols, particularly trimethylolpropane, to corresponding hydroxycarboxylic acids, such as 2,2-bis(hydroxymethyl)butyric acid, under moderate conditions with high selectivity and high yield (3), while several microorganisms, for example, *Gluconobacter oxydans*, which is used for oxidation of straight diols and polyols, show no ability to oxidize branched polyols (data not shown). To further study the oxidative biotransformation capabilities of *Corynebacterium* sp. (ATCC 21245), identification of the genes encoding different proteins, including the enzymes responsible for the oxidation of polyols, is required.

The genomic DNA was extracted from pure culture using the ZR Fungal/Bacterial miniprep kit (Zymo Research), and then a complete-genome sequence was achieved by Pacific Bioscience (PacBio) long-read sequencing using 20-kb SMARTbell libraries (Macrogen, South Korea). All generated sequence reads (192,756) were assembled into six contigs using HGAP software version 3, and the G+C content was calculated for all contigs. Genome assembling shows that the genome size of *Mycobacterium* sp. MS1601 totals 6,829,132 bp, including one chromosome with a size of 6,407,860 bp. Then, coding sequences (CDSs), tRNA genes, and rRNA genes were annotated automatically using Prokka (http://www.vicbioinformatics.com/software.prokka.shtml). Locations, functions, nucleotide sequences, and amino acid sequences for the different proteins in all of the contigs were determined using the RAST server version 2 (http://rast.nmpdr.org) and the NCBI Prokaryotic Genome Annotation Pipeline. The annotation results identified 6,620 CDSs, 58 tRNAs, and six rRNAs. Moreover, the number of genes encoding different dehydrogenases and oxidoreductases were 428 and 152, respectively. We also found 25 genes encoding alcohol dehydrogenase and 41 genes for aldehyde dehydrogenase enzymes in different locations on the bacterial genome. According to genome comparison results obtained from RAST, the closest genomes to *Corynebacterium* sp. (ATCC 21245) are those for *Mycobacterium vanbaalenii* PYR-1, *Mycobacterium vanbaaleni vanbaalenii* PYR-1, and *Mycobacterium* sp. MCS, which are used widely in the degradation of aromatic polycyclic hydrocarbons (4–7). All of these species are soil-inhabiting, fast-growing, and nonpathogenic mycobacteria.

In order to confirm the reclassification of *Corynebacterium* sp. ATCC 21245 to *Mycobacterium* sp. MS1601, 16S rRNA gene analysis was performed, and the obtained results showed >99% homology to the *Mycobacterium* genus.

The complete genome sequence of *Mycobacterium* sp. MS1601 will facilitate the identification, characterization, and evolution of oxidative enzymes and provide basic information for the wider exploitation of selective oxidation of polyols.

### Accession number(s).

The complete genome sequence of *Mycobacterium* sp. MS1601 has been deposited in DDBJ/EMBL/GenBank under the accession numbers CP019420 to CP019425. This strain is available as *Corynebacterium* sp. (ATCC 21245) from ATCC.
